# Enhanced Antimicrobial Activity of N-Terminal Derivatives of a Novel Brevinin-1 Peptide from The Skin Secretion of *Odorrana schmackeri*

**DOI:** 10.3390/toxins12080484

**Published:** 2020-07-30

**Authors:** Xiaowei Zhou, Yue Liu, Yitian Gao, Yuanxing Wang, Qiang Xia, Ruimin Zhong, Chengbang Ma, Mei Zhou, Xinping Xi, Chris Shaw, Tianbao Chen, Di Wu, Hang Fai Kwok, Lei Wang

**Affiliations:** 1 Department of Nutrition, Henry Fok School of Food Science and Engineering, Shaoguan University; Shaoguan 512005, China; xzhou06@qub.ac.uk (X.Z.); sgu_zrm@sgu.edu.cn (R.Z.); 2 Institute of Translational Medicine, Faculty of Health Sciences, University of Macau, Avenida da Universidade, Taipa, Macau SAR; 3 Natural Drug Discovery Group, School of Pharmacy, Queen’s University, Belfast BT9 7BL, Northern Ireland, UK; yliu58@qub.ac.uk (Y.L.); c.ma@qub.ac.uk (C.M.); m.zhou@qub.ac.uk (M.Z.); chris.shaw@qub.ac.uk (C.S.); t.chen@qub.ac.uk (T.C.); l.wang@qub.ac.uk (L.W.); 4 College of Life and Environmental Science, Wenzhou University, Wenzhou 325035, China; gyt@wzu.edu.cn; 5 State Key Lab of Food Science and Technology, Nanchang University, Nanchang 330047, China; yuanxingwang@ncu.edu.cn; 6 Department of Food Science and Engineering, Key Laboratory of Animal Protein Food Processing Technology of Zhejiang Province, College of Food and Pharmaceutical Sciences, Ningbo University, Ningbo 315832, China; xiaqiang@nbu.edu.cn; 7 Chemical Biology Research Centre, School of Pharmaceutical Science, Wenzhou Medical University, Wenzhou 325035, China; wudi2017@wmu.edu.cn

**Keywords:** skin-derived peptides, antimicrobial activity, stability, waxworm model

## Abstract

Antimicrobial peptides (AMPs) are promising therapeutic alternatives compared to conventional antibiotics for the treatment of drug-resistant bacterial infections. However, the application of the overwhelming majority of AMPs is limited because of the high toxicity and high manufacturing costs. Amphibian skin secretion has been proven to be a promising source for the discovery and development of novel AMPs. Herein, we discovered a novel AMP from the skin secretion of *Odorrana schmackeri*, and designed the analogues by altering the key factors, including conformation, net charge and amphipathicity, to generate short AMPs with enhanced therapeutic efficacy. All the peptides were chemically synthesised, followed by evaluating their biological activity, stability and cytotoxicity. OSd, OSe and OSf exhibited broad-spectrum antibacterial effects, especially OSf, which presented the highest therapeutic index for the tested bacteria. Moreover, these peptides displayed good stability. The results from scanning electron microscopy and transmission electron microscopy studies, indicated that brevinin-OS, OSd, OSe and OSf possessed rapid bactericidal ability by disturbing membrane permeability and causing the release of cytoplasmic contents. In addition, OSd, OSe and OSf dramatically decreased the mortality of waxworms acutely infected with MRSA. Taken together, these data suggested that a balance between positive charge, degrees of α-helicity and hydrophobicity, is necessary for maintaining antimicrobial activity, and these data successfully contributed to the design of short AMPs with significant bactericidal activity and cell selectivity.

## 1. Introduction

Recently, the emergence of “superbug” bacteria caused by the abuse of conventional antibiotics, has become a serious public health challenge. Therefore, the development of novel biomolecules that possess significant antibacterial activity and are less likely to provoke resistance, is urgently required.

Antimicrobial peptides (AMPs) have long been thought to have a promising role in the fight against multidrug-resistant bacteria due to their unique bactericidal mechanism and broad-spectrum activity [1,2,3]. Recently, the application of AMPs as commercial therapeutics has been extensively researched [4,5]. Although AMPs are promising for clinical application, they are facing enormous obstacles to be drug candidates [6], including low metabolic stability [7], high manufacturing cost [8,9], safety [10] and strict supervision by the regulatory agencies of government [11]. With the purpose of improving antimicrobial activity and physiological stability as well as reducing cytotoxic effects, a variety of changes, such as glycosylation, fluorination, cyclisation, replacement of specific amino acids, truncation of original peptides and the incorporation of D- and non-natural amino acids, have been applied [12,13].

Brevinin-1 peptides, an AMP family from the skin secretions of the Ranidae family of frogs, usually possess a broad-spectrum antibacterial activity [14]. Normally, brevinin-1 peptides contain 24 amino acids with an invariant Pro residue at the 14th position from the N-terminal and a Ranabox (Cys^18^-(Xaa)4-Lys-Cys^24^) at the C-terminal [15]. However, most have strong haemolytic activity [16,17,18,19,20] because of their high hydrophobicity. Previous research reported that the disulfide bridge is not necessary for strong antimicrobial efficacy and the Ranabox at the C-terminal, being transported to the centre of peptides, reduces haemolytic activity without affecting antibacterial properties [21,22]. Meanwhile, the C-terminal Ranabox region plays a crucial role in targeting membranes [23]. However, the effect of the N-terminal region of brevinin-1 peptides has not been studied.

Therefore, in this study, a novel brevinin-1 peptide, brevinin-1OS, was isolated from the skin secretion of *Odorrana schmackeri*. For further investigation of the structure-activity relationships of this peptide and optimisation of its physiochemical properties, six synthetic peptides were designed from its template. The peptides were synthesised, and the purified peptides were subjected to antimicrobial activity assays, haemolysis activity assays and antibiofilm activity assays. The secondary structures of peptides were predicted by I-TASSER software and tested by circular dichroism (CD). The stability of peptides in high salt, at high temperature and in serum, was also examined. Their interactions with bacterial membranes were validated by dye-staining and microscopy. The pig skin model was applied to evaluate the bacterial biofilm antimicrobial ability of OSf. The in vivo studies of peptides were performed by use of the bacterial-infected *Galleria mellonella* larvae model.

## 2. Results

### 2.1. Peptide Identification, Design and Conformational Studies

**Figure A1:**
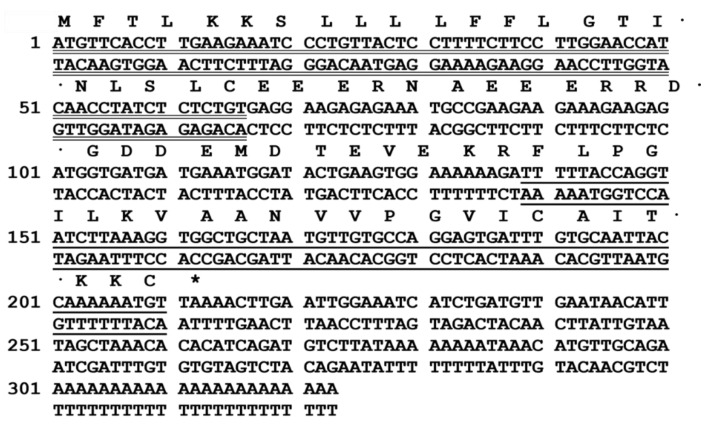
Nucleotide and corresponding translated open-reading frame of precursor cDNA cloned from the skin secretion of *Odorrana schmackeri* cDNA library that encodes brevinin-1OS (Genbank accession number: MK922297). The biosynthetic precursor consists of a putative signal peptide domain (double underline) and a mature peptide domain (single underline). The stop codon is indicated by an asterisk.

**Figure A2:**
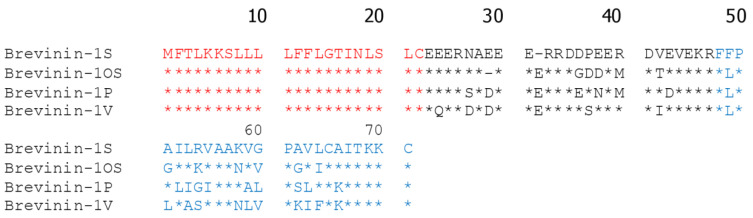
Alignments of the amino acid sequences of the biosynthetic precursors of brevinin-1 family members that were discovered from Ranidae frogs. Identical amino acids are indicated by the asterisks. Putative signal peptide domain and mature peptide domain are indicated by red and blue, respectively. The gap in brevinin-1OS is introduced to maximize the length and optimise the identities. The gaps (_) are used to maximise alignments.

**Figure A3:**
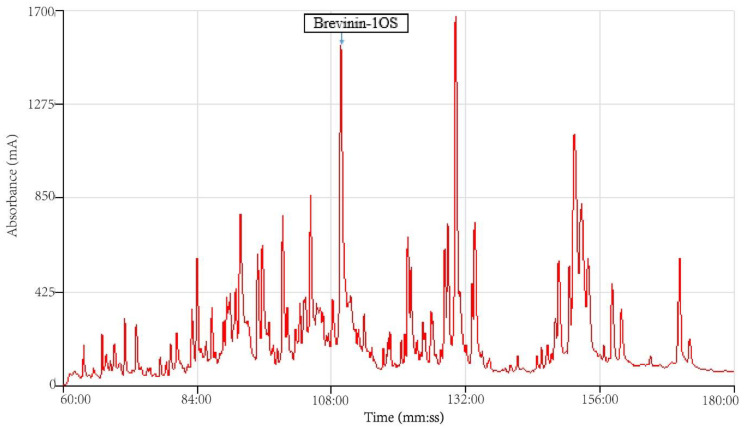
The region of RP-HPLC Chromatogram of the skin secretion of *Odorrana schmackeri*. The absorbance was detected at the wavelength of 214 nm. The elution peak of brevinin-1OS is indicated by an arrow.

**Figure A4:**
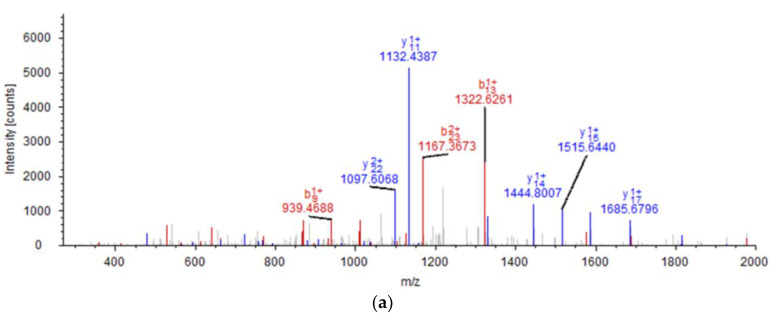

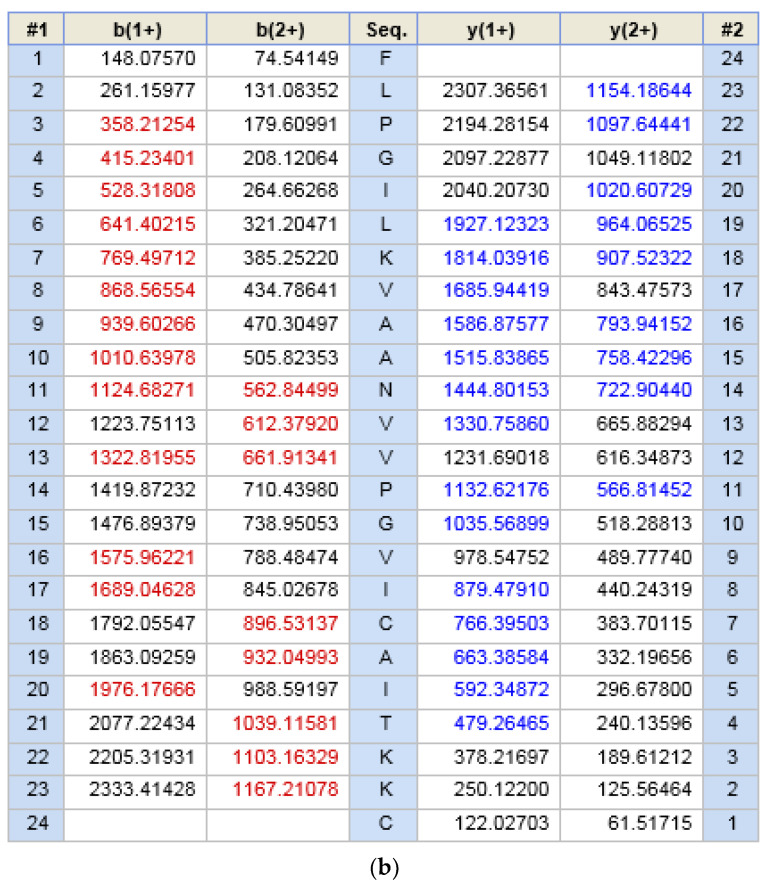
The identification of brevinin-1OS in the RP-HPLC fraction isolated from the skin secretion of *Odorrana schmackeri*. (**a**) The MS/MS spectra of brevinin-1OS isolated from the skin secretion. The observed b- and y- fragment ions are annotated by red and blue. (**b**) The list of theoretical singly and doubly charged b- and y-ions of brevinin-1OS. The matched ions during the search via Sequest algorithm of Protein Discoverer 1.0 were coloured in the table.

**Figure A5:**
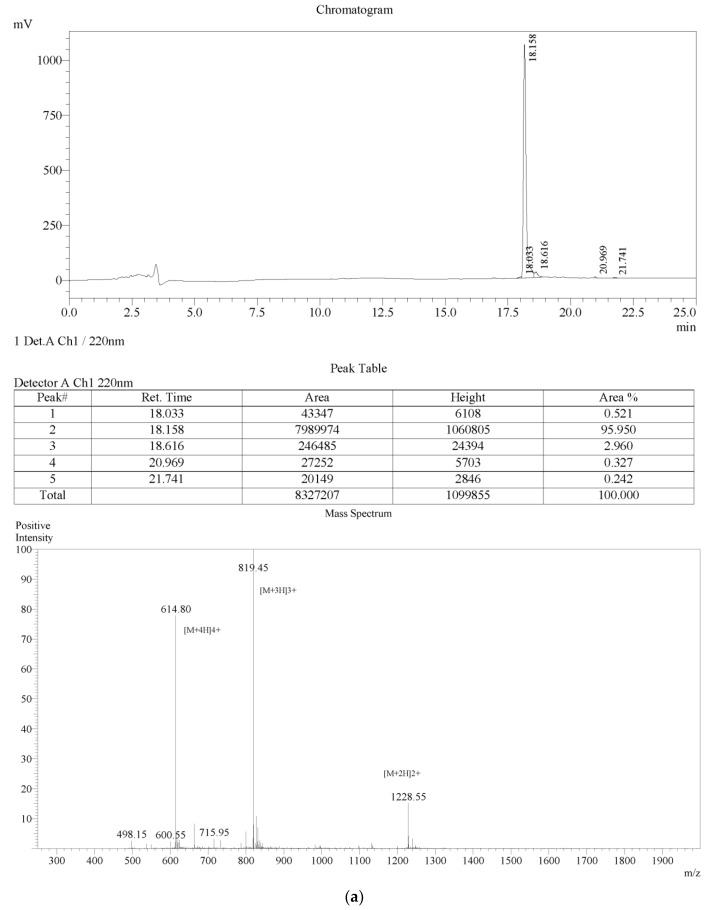

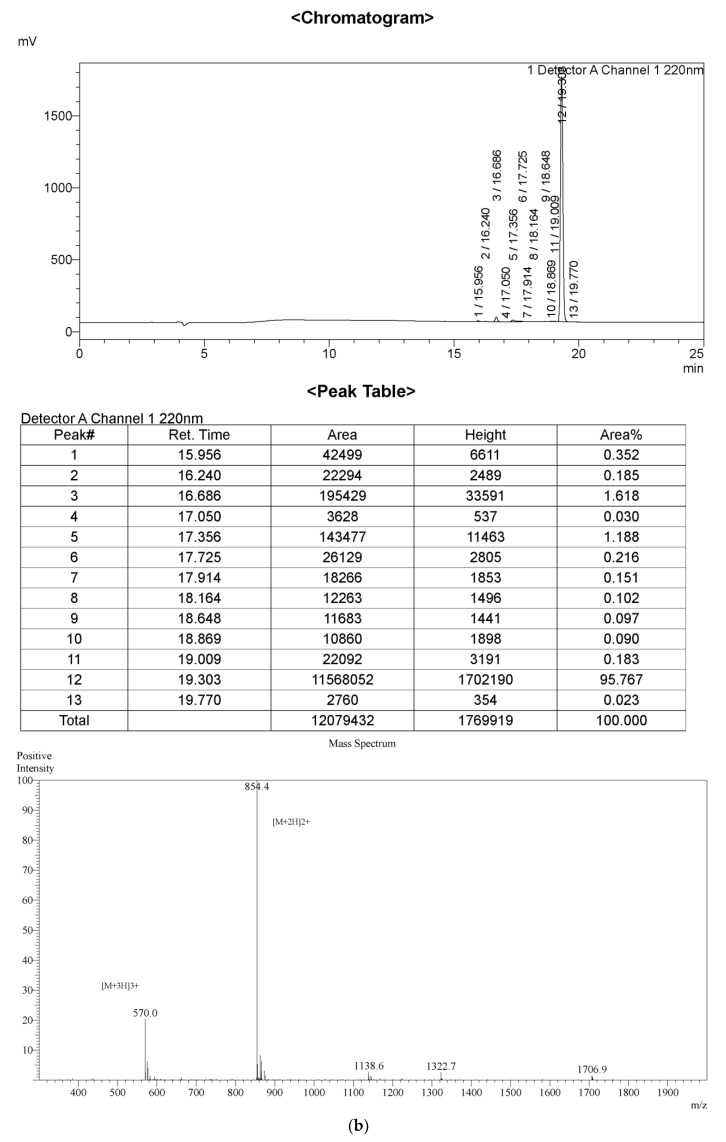

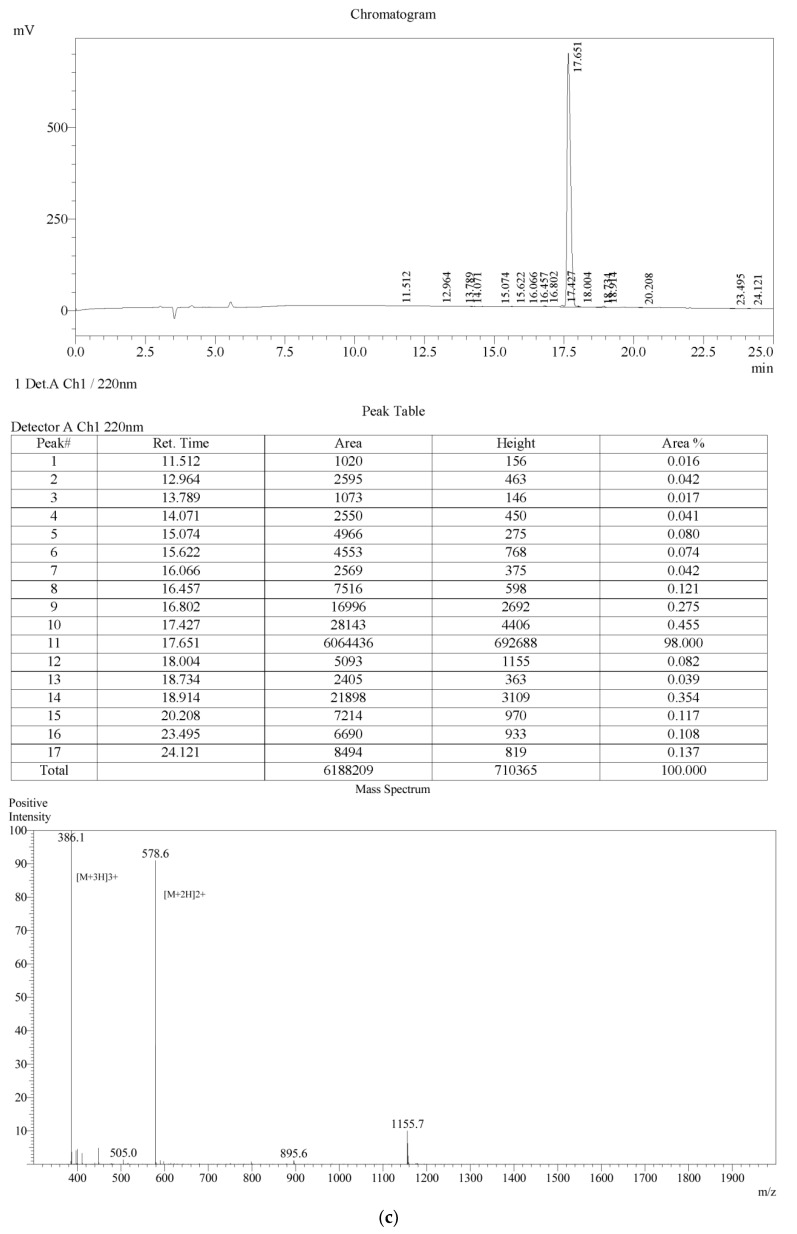

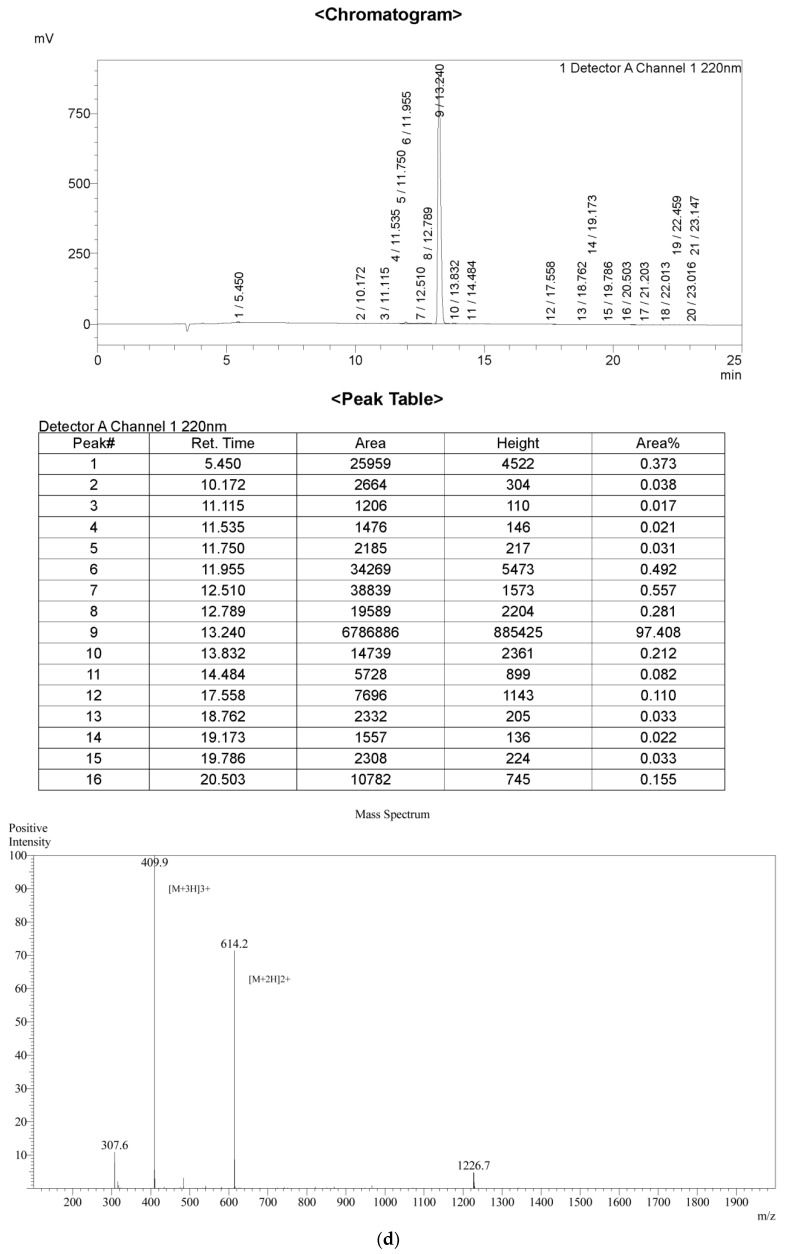

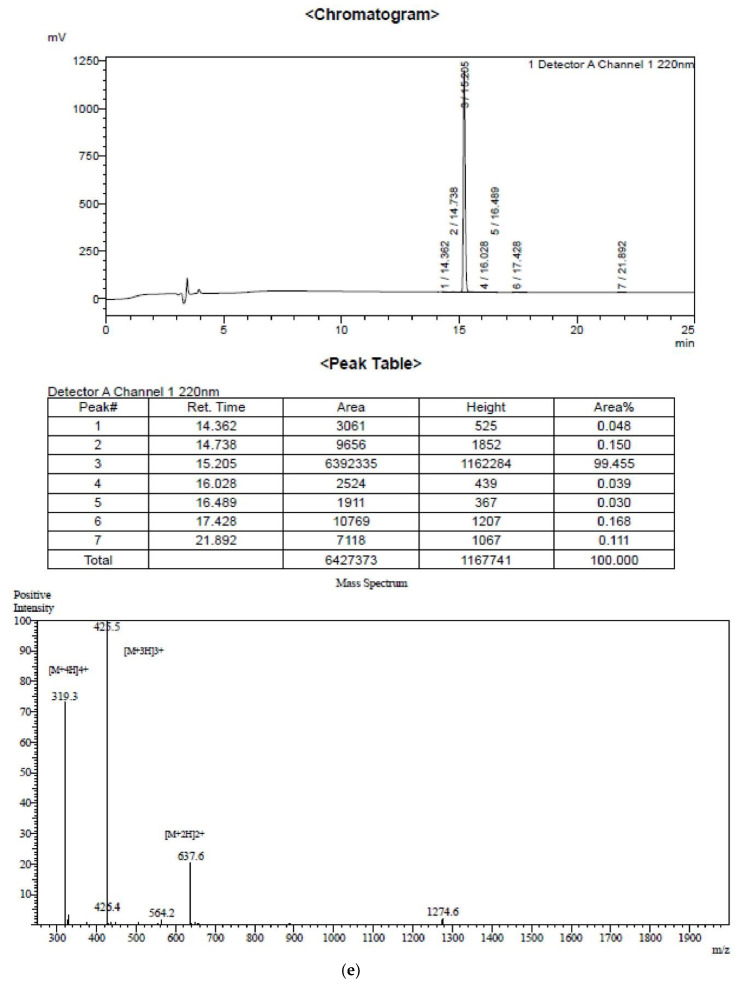

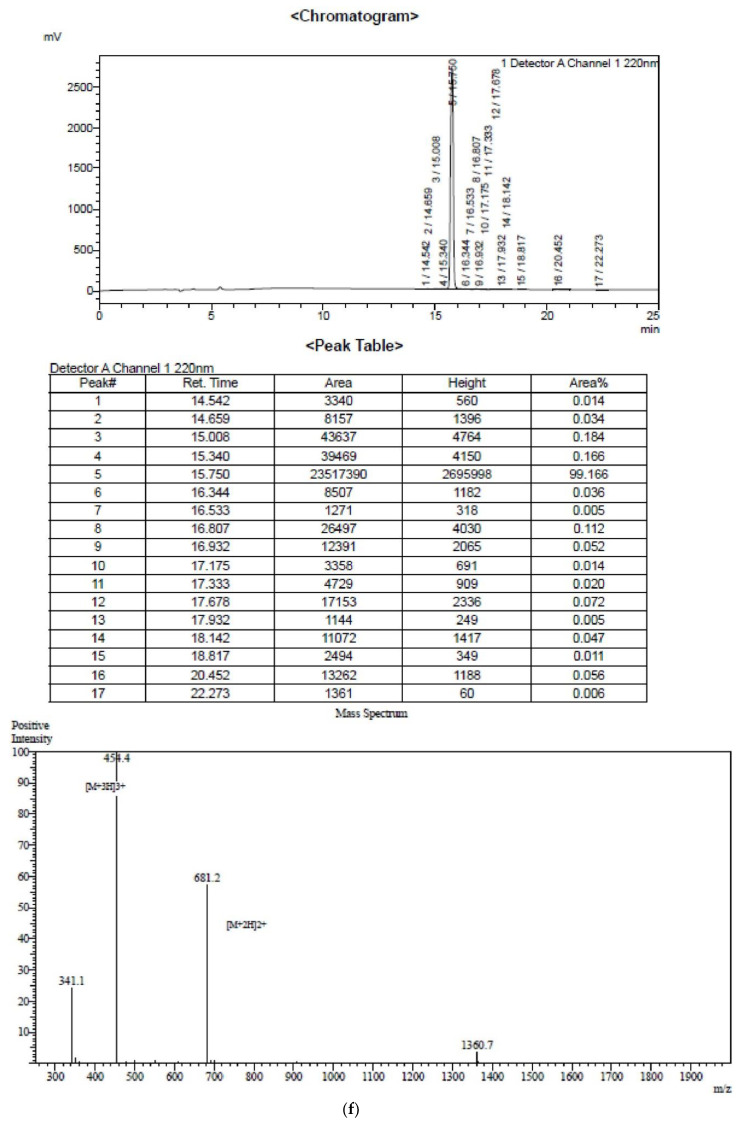

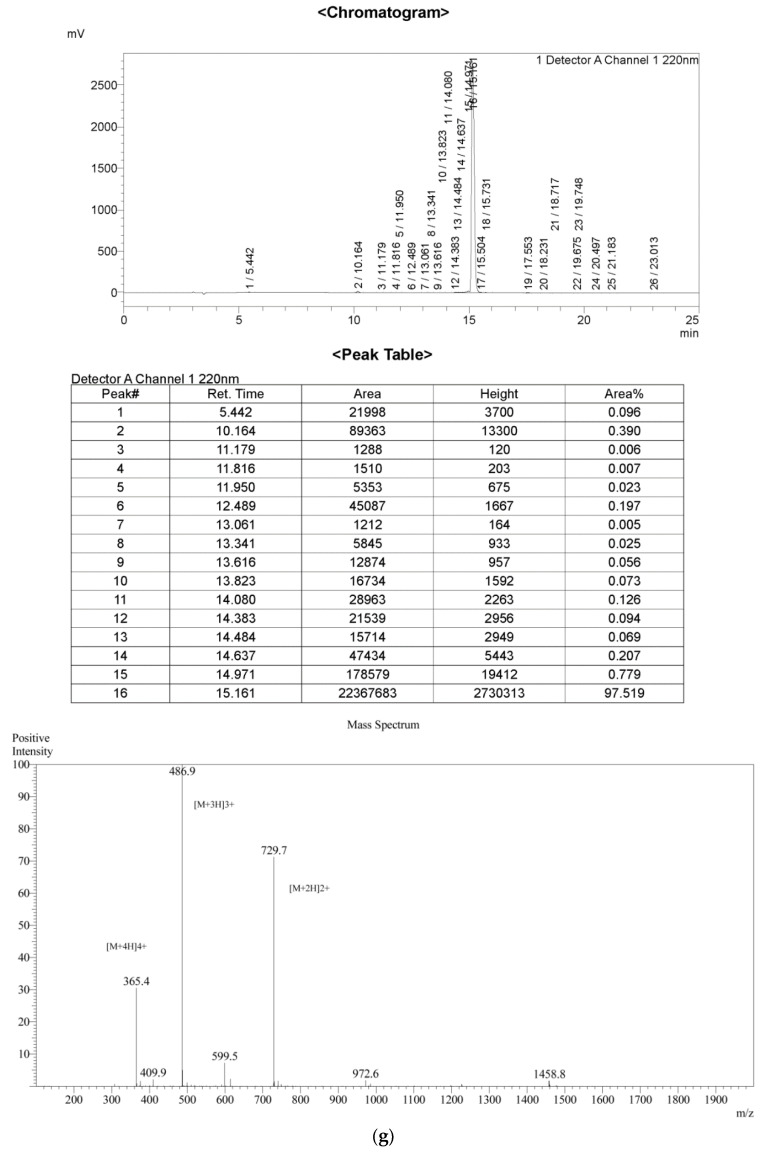
The examination of the purity of the peptides using RP-HPLC and mass spectrometer. The chromatogram and MS spectra indicate that the purity of Brevinin-1OS (**a**), OSa (**b**), OSb (**c**), OSc (**d**), OSd (**e**), OSe (**f**) and OSf (**g**) are more than 95%.

The cDNA encoding precursor sequence of brevinin-1OS was shown in Figure A1. Additionally, brevinin-1OS showed high similarity to other brevinin-1 peptides (Figure A2). Brevinin-1OS was successfully isolated and identified in the skin secretion of *Odorrana schmackeri* (Figure A3 and Figure A4). Based on the structure of brevinin-1OS, six analogues derived from the N-terminal domain were designed. All the peptides were chemically synthesised by solid-phase synthesis, and further purified by RP-HPLC and analysed by mass spectrometry (Figure A5).

**Figure A6:**
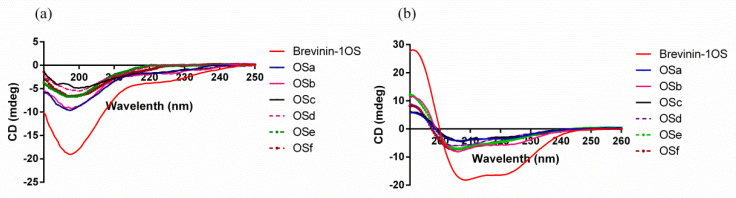
CD spectra of brevinin-1OS and its analogues in 10 mM NH_4_AC solution (**a**) and in 50% TFE in 10 mM NH_4_AC solution (**b**).

**Figure A7:**
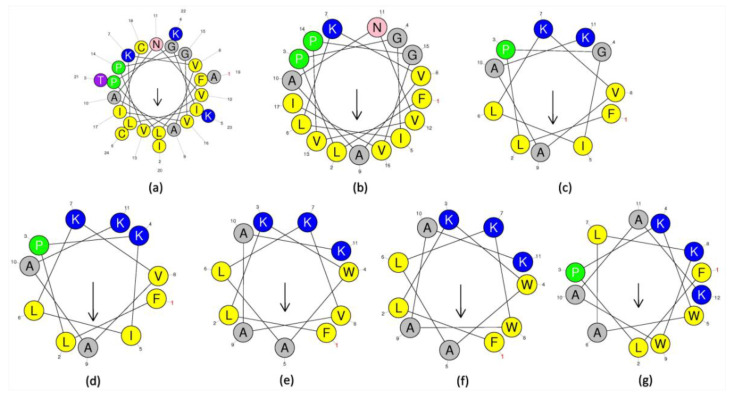
Heliquest wheel projects of brevinin-1OS and its analogues: (**a**) brevinin-1OS; (**b**) OSa; (**c**) OSb; (**d**) OSc; (**e**) OSd; (**f**) OSe and (**g**) OSf.

To further identify the secondary structure of purified peptides, they were dissolved in 10 mM ammonium acetate (NH_4_AC) and 50% trifluoroethanol (TFE) in 10 mM NH_4_AC, respectively, to achieve the concentration of 50 µM. This revealed that peptides formed typical α-helical structures in the TFE solution, and random coils in the NH_4_AC solution (Figure A6). The circular dichroism (CD) data of the peptides in TFE solution was submitted to the K2D3 webserver to analyse the α-helical content. Moreover, helical wheel projections of brevinin-1OS and its analogues were conducted (Figure A7) and the physicochemical properties of all peptides are summarized in Table 1. The results revealed that brevinin-1OS possessed 94.61% α-helicity but the α-helicity of the modified analogues was decreased dramatically.

### 2.2. Antimicrobial, Antibiofilm and Haemolytic Activity of Brevinin-1OS and Its Analogues

**Figure A8:**
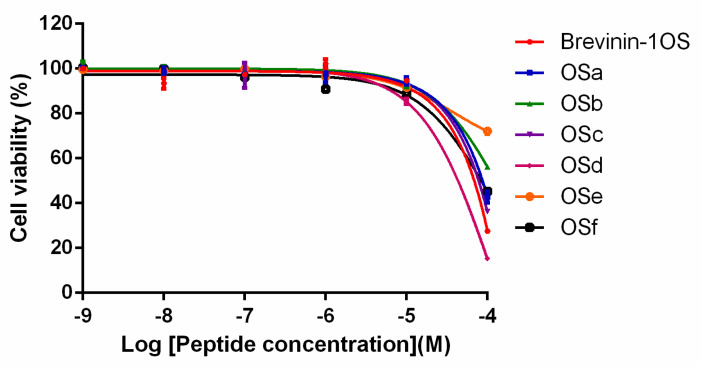
The cell viability of human dermal microvascular endothelium cells (HMEC) when treated with various concentrations of Brevinin-1OS (red), OSa (blue), OSb (green), OSc (purple), OSd (grey), OSe (orange) and OSf (Black).

The antimicrobial activity of brevinin-1OS and its analogues are shown in Table 2. It was clearly revealed that brevinin-1OS possessed significant activity against Gram-positive bacteria and the yeast *Candida albicans* (*C. albicans*), while it exhibited weak activity against Gram-negative bacteria. Among the designed peptides, OSa had no antimicrobial activity against selected microorganisms. OSb and OSc only retained mild antimicrobial activity against *Staphylococcus aureus* (*S. aureus*) and *C. albicans*. Interestingly, OSd, OSe and OSf presented broad-spectrum antibacterial properties. The peptides presented different haemolytic activity against horse red blood cells with the order: OSe > 1OS > OSf > OSb > OSa ≈ OSc ≈ OSd (Table 2). OSd showed the highest therapy index (TI) value among these peptides. Besides, all the peptides exhibited the cytotoxicity against human dermal microvascular endothelium cell line (HMEC-1) at 100 µM (Figure A8).

As brevinin-1OS, OSd, OSe and OSf showed significant antimicrobial activity against relevant bacteria, they were subjected to the anti-biofilm assays. The results demonstrated that the four peptides were able to inhibit the formation of the biofilms of the selected strains and eradicate the mature biofilms of *S. aureus*, Meticillin-resistant *Staphylococcus aureus* (MRSA) (Table 3). Remarkably, OSd, OSe and OSf were capable of disrupting the *Enterococcus faecalis* (*E. faecalis*) biofilm.

### 2.3. Antimicrobial Activity in Different Environmental Conditions

Considering that the antimicrobial activity of AMPs could be influenced to a certain extent by the different microenvironment condition in the body fluids, such as salts, pH and serum, which dramatically limits their clinical applications [24,25]. Additionally, pharmaceutical manufacturing may involve the processes at different temperatures (e.g., sterilization of drugs). Besides, denaturation of protein molecules at high temperature is the common feature that could inactivate the bioactivity irreversibly. Therefore, the antibacterial activity of our peptides against six organisms under several biologically-relevant conditions and different temperature, was tested. As shown in Table 4, the MICs of peptides increased at least two-fold in the presence of salts. pH had a greater effect on the MIC values of the peptides. Brevinin-1OS and three designed peptides also retained significant antimicrobial activity in the presence of serum. The results also showed that the peptides were tolerant of high temperatures.

### 2.4. Outer Membrane (OM) and Inner Membrane (IM) Permeability Assays

**Figure 1:**
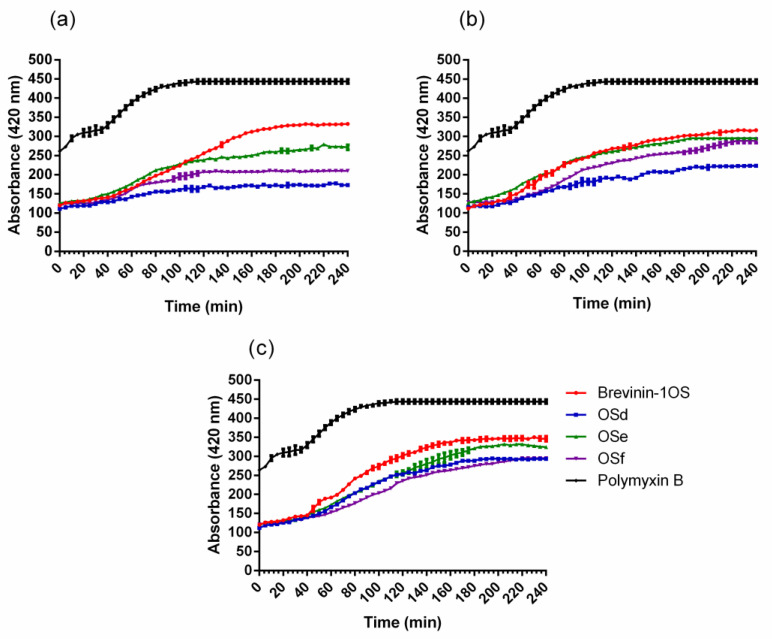
The outer membrane (OM) permeabilisation assays of *E.coli* in the treatment of the peptides at (**a**) 1 × MICs, (**b**) 2 × MICs and (**c**) 4 × MICs. Fluorescence intensity enhanced by the uptake of 1-*N*-phenylnaphthylamine (NPN) in the OM was monitored. The polymyxin B (10 µg/mL) was employed as a positive control. Data represent means ± standard error of the mean of three independent experiments.

Due to brevinin-1OS, OSd, OSe and OSf showing significant antimicrobial activity against *E. coli*, OM and IM permeability assays were performed to determine the integrity of OM and plasma cell membrane of *E. coli* after the treatment with peptides. The results of OM permeability revealed that all peptides could permeabilise the *E. coli* OM at concentrations ranging from 1 × MIC to 4 × MIC (Figure 1).

**Figure 2:**
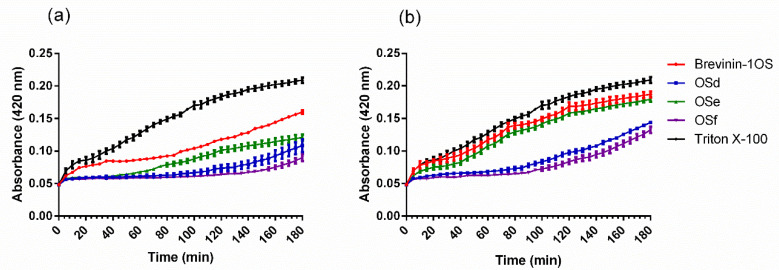
Permeability of the inner membrane (IM) in the treatment of the peptides at (**a**) 1 × MICs and (**b**) 4 × MICs. Hydrolysis of *o*-Nitrophenyl-β-D-galactopyranoside (ONPG) due to the release of cytoplasmic β-galactosidase from the compromised cell membrane of *E.coli*. The absorbance of *o*-Nitrophenyl cleaved from OPNG was determined spectroscopically under 420 nm. The Triton X-100 was used as a positive control. Data represent means ± standard error of the mean (*n* = 3).

Similarly, all of the peptides showed dose-dependent IM permeabilisation (Figure 2). Specifically, β-galactosidase was released from *E. coli* cells with a lag time of about five min when treated with different concentrations of peptides, however, the relative fluorescence that reached steady state differed at different concentrations of peptides. The higher the concentration of peptides used, the earlier a steady state could be reached. The obvious IM permeability order was OSe > OSf > OSd.

### 2.5. Scanning Electron Microscopy (SEM)

**Figure 3:**
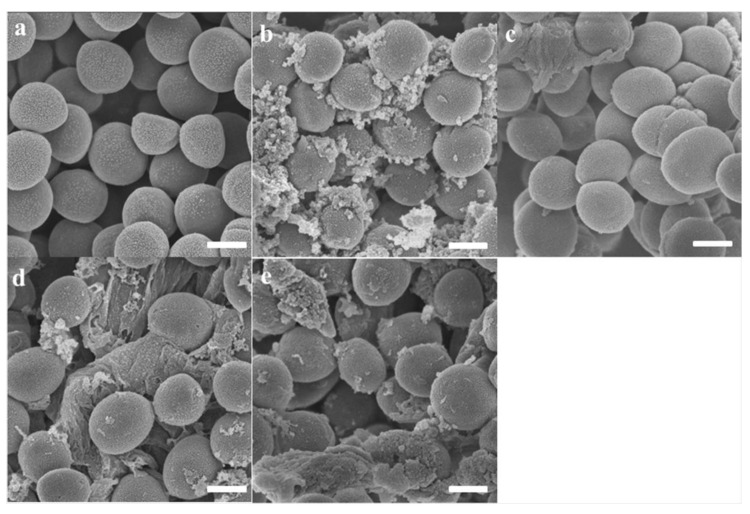
SEM micrographs of MRSA treated with brevinin-1OS at 1 × MICs for 60 min. (**a**) untreated MRSA cells; (**b**) Brevinin-1OS, (**c**) OSd, (**d**) OSe and (**e**) OSf treated MRSA cells. Scale bar = 500 nm.

**Figure 4:**
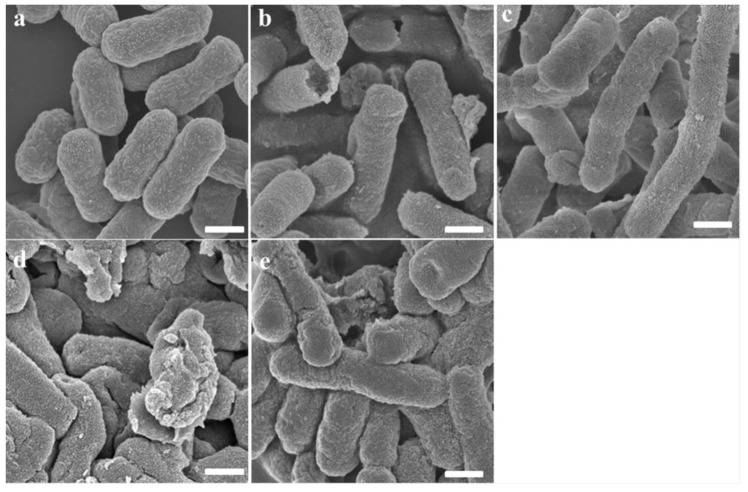
SEM micrographs of *E. coli* treated with peptides at 1 × MICs for 60 min. (**a**) untreated *E. coli* cells; (**b**) Brevinin-1OS, (**c**) OSd, (**d**) OSe and (**e**) OSf treated *E. coli* cells. Scale bar = 1 µm.

The integrity and micromorphology of bacterial cells were assessed by SEM. As shown in Figure 3 and Figure 4, the control cells contained smooth, integral and full membranes, however, the MRSA cells were damaged dramatically after treatment with peptides. Specifically, MRSA cells treated with peptides displayed distinct irregularities, blisters and breakages. In addition, the partial *E. coli* cells surface exposed to the peptides revealed membrane distortion, roughness and corrugation.

### 2.6. Transmission Electron Microscopy (TEM)

**Figure 5:**
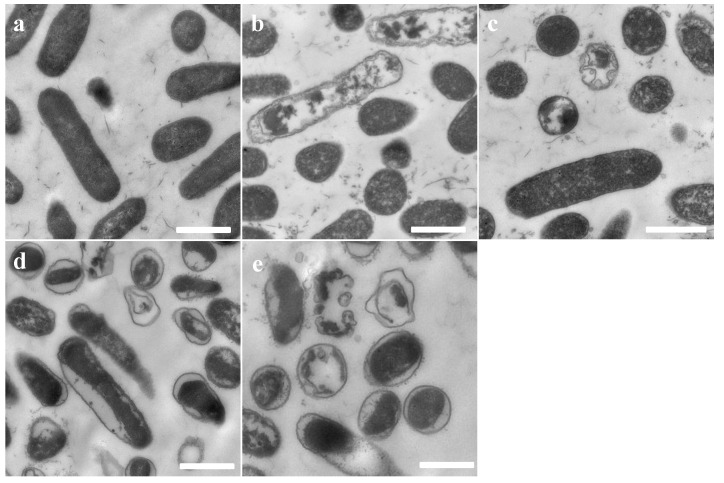
TEM micrographs of *E. coli*: (**a**) Untreated *E. coli* cells; (**b**) Brevinin-1OS, (**c**) OSd, (**d**) OSe and (**e**) OSf treated *E. coli* cells. Scale bar = 1 µm.

**Figure 6:**
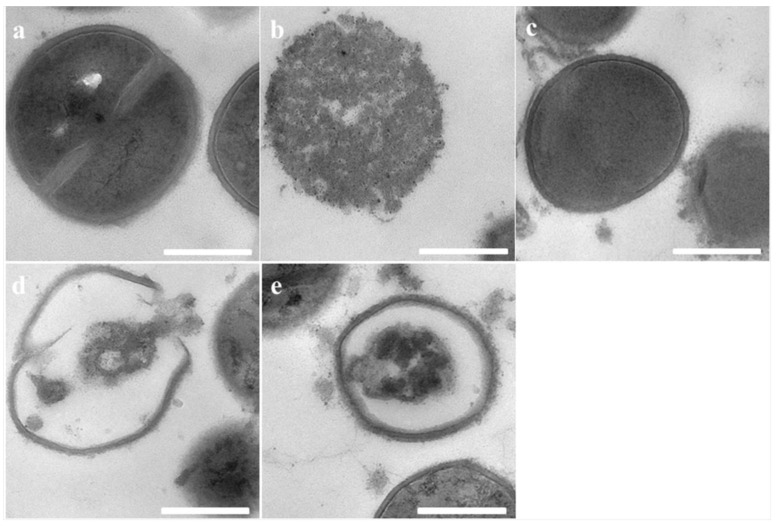
TEM micrographs of MRSA: (**a**) Untreated MRSA cells; (**b**) Brevinin-1OS, (**c**) OSd, (**d**) OSe and (**e**) OSf treated MRSA cells. Scale bar = 500 nm.

Transmission electron microscopy was further conducted to study the morphological character and intracellular alteration of *E. coli* and MRSA upon treatment with peptides. Untreated *E. coli* and MRSA cells retained perfect smooth surfaces and dense internal structures, however, significant morphological alterations were observed in the TEMs of *E. coli* and MRSA cells treated with the peptides. As shown in Figure 5 and Figure 6, brevinin-1OS, OSd, OSe and OSf could destroy bacterial membranes dramatically. Specifically, both were able to induce the corruption and uneven intracellular reduction of *E. coli* cells. In addition, after treatment with peptides for 1 h, large numbers of *E. coli* cells showed a significant detachment of the outer membrane and inner membrane as in Figure 5. Additionally, compared with OSd, OSe and OSf probably demonstrated greater destructive ability against MRSA cells. As shown in Figure 6, the cytoplasm of MRSA cells was almost completely released by treatment with OSe and OSf, however, the cytoplasm of MRSA cells treated with OSd was released as briquettes.

### 2.7. Time-Killing Assay Against MRSA

**Figure 7:**
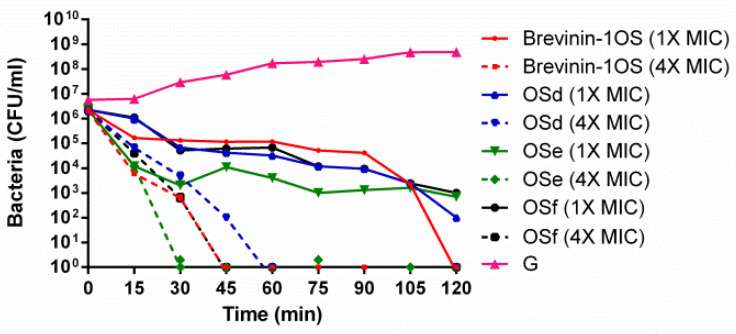
Time killing curve of MRSA by brevinin-1OS, OSd, OSe and OSf at different concentrations. G represents growth control. Data represent means ± standard error of the mean of three independent experiments.

In this study, native brevinin-1OS and three short peptide analogues, including OSd, OSe and OSf which possessed significant antimicrobial effects against MRSA, were selected. The killing effect of peptides was investigated at concentrations corresponding to the MIC and 4 × MIC. The time-killing curve revealed that brevinin-1OS, OSd, OSe and OSf at a concentration of 4 × MIC caused the complete killing of MRSA within 45 min, 60 min, 30 min and 45 min, respectively (Figure 7). OSe showed the most rapid killing kinetic ability against MRSA compared with OSd and OSf, while, similar rapid killing kinetics were observed with OSd and OSf against MRSA.

### 2.8. Skin Disinfection Test of Peptides

**Figure 8:**
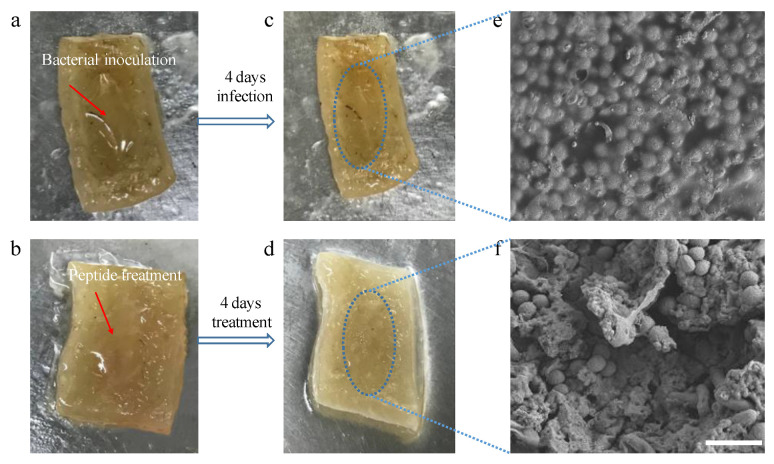
Flow charts of MRSA infection on pig skin, disinfection by OSf and corresponding SEM photographs of wound: (**a**) Images of the inoculation of MRSA on the pig skin; (**b**) images of the OSf treated wound after inoculation of MRSA; (**c**,**d**) images of morphologies of the infected and uninfected pig skin tissues, respectively; (**e**,**f**) SEM Photographs of MRSA-infected and disinfected wounds, respectively. Scale bar = 10 µm.

The pig skin model was conducted to investigate the disinfecting properties in vitro because of more suitable and more reliable properties than agar plate models. OSf was selected as an example in this assay. As shown in Figure 8, the surface of the infection wound showed lots of yellow spots, however, after four days treatment of OSf, a clearance of the surface of the pig skin was observed in Figure 8d, suggesting that effective anti-infection had occurred with OSf. Moreover, as shown in Figure 8e, and Figure 8f, supporting this, in the infected wound, large amounts of MRSA were accumulated on the surface of skin tissue, and a thick biofilm was observed. Nevertheless, the MRSA cells that accumulated on the wound were almost eradicated (Figure 8f). Specifically, the MRSA morphology was altered dramatically as virtually no unbroken MRSA cells were observed.

### 2.9. Treatment of MRSA-Infected Galleria Mellonella Larvae with Peptides

**Figure 9:**
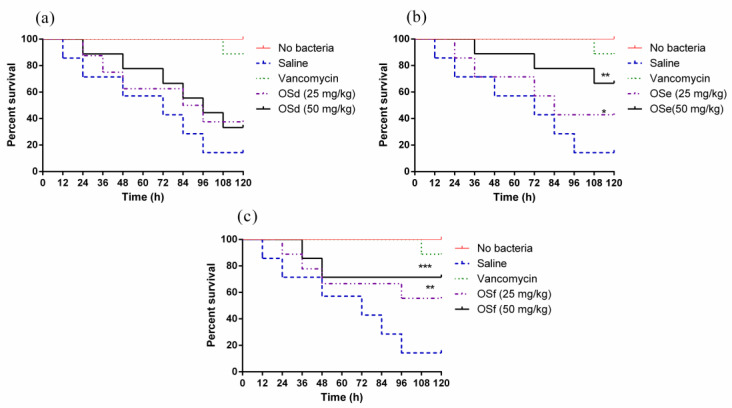
The percentage survival of the larvae infected with MRSA. They were treated with OSd (**a**), OSe (**b**) and OSf (**c**), respectively. The infected larvae treated with 20 mg/kg of vancomycin (green dotted line) is a positive control. The significance of the effects of peptides was analysed using log-rank test and indicated as *** *p* < 0.001, ** *p* < 0.01 and * *p* < 0.05.

**Figure A9:**
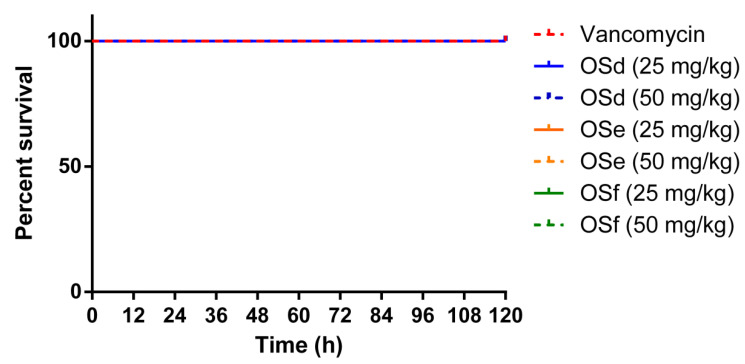
Larvae without any MRSA infection were injected with peptides and vancomycin respectively to access the cytotoxicity of the drugs to larvae bodies. No noticeable cytotoxicity was observed among these control groups. Effect of treatment of *G. mellonella* larvae (*n* = 30 for each group) without any infection and only treated with 5 µL vancomycin (20 mg/kg), OSd, OSe and OSf.

The mortality of MRSA-infected larvae after peptide treatments is shown in Figure 9. Specifically, OSe and OSf reduced the mortality of infected larva. In addition, the survival rates of larvae treated with 50 mg/kg of OSe and OSf were higher than that of waxworms treated with 25 mg/kg of peptides. All of the peptides were not toxic to the larvae (Figure A9). Notably, OSf showed the highest effect on reducing the mortality of infected larvae among the three peptides.

## 3. Discussion

This study identified a novel brevinin-1 peptide, brevinin-1OS, from the skin secretion of *Odorrana schmackeri*, which demonstrates several common structural characteristics. Firstly, like brevinin-1 [26], the N-terminal domain of brevinin-1OS consists of a large proportion of hydrophobic amino acid residues. Moreover, it contains a Ranabox at the C-terminus, which is a disulphide-bridged cyclic heptapeptide (Cys^18^-(Xaa)4-Lys-Cys^24^) [15]. Additionally, brevinin-1OS is cationic and has four residues at the conserved positions (Ala^9^, Cys^18^, Lys^23^, Cys^24^), which are known as invariable residues for brevinin-1 [27]. Besides, Pro^14^, a key residue that produces a stable kink in the molecule [28], also exists in brevinin-1OS.

Brevinin-1OS presented significant antimicrobial activity against Gram-positive bacteria and fungi. However, it also possessed high haemolytic which may severely limit the potential for therapeutic use. The data revealed that OSa was not observed to have any antimicrobial activity and haemolytic activity, which is consistent with the previous research [23,29]. It is assumed that the elimination of bioactivity may be caused by the decrease in the net charge after excising the Ranabox. As previously reported, the net positive charge of at least +2 is essential for the antimicrobial action of cationic peptides [30]. Our data suggested that the cationic amino acids in the Ranabox of brevinin-1 peptides and N-terminal domain of brevinin-1 peptides, might be equally responsible for the function of brevinin-1 peptides rather than the Ranabox alone. Moreover, our results revealed that the activity of OSb and OSc were slightly enhanced on Gram-positive bacteria because of the addition of cationic amino acid. However, the MIC values of OSc was still low. We speculated that it may be due to the existence of Pro^3^ in OSc, which is the strongest structural disruptor in the middle of formal secondary structural elements [31]. Therefore, we removed Pro and introduced Trp to the sequence. In the meantime, in order to optimize the cationic and hydrophobic surfaces showed in the helical wheel projection (Figure A7), the sequence was modified for the design of OSd. Notably, the antimicrobial activity of OSd was dramatically enhanced compared to OSc. This observation was supported by previous studies that Trp residue has an indole side chain which can anchor to the polar-apolar interface and this feature gives tryptophan an inherent membrane interactive ability [32,33]. At the same time, removal of Pro could allow OSd to form more α-helix content than OSc, which was in accordance with the determination of α-helicity in 50% TFE solution (Table 1). With this significant improvement, the Val of OSd was further substituted by Trp for the design of OSe, which did exhibit stronger antimicrobial activity than OSd. Based on wheel-helix and CD spectra results (Figure A6 and Figure A7), OSd showed a more ideal α-helical structure than OSe or OSf, while OSd is weaker than both in inhibiting the growth of microorganisms. This phenomenon suggested that the peptide α-helical structural characteristics may play a key factor in bacterial cell death, but that the amount of α-helical content may not have a positive correlation with antibacterial activity [34].

Interestingly, our data showed that the hydrophobicity of OSe was in between OSd and OSf, however, OSe exhibited the strongest antibacterial activity among these three peptides. The existence of a threshold of hydrophobicity related to the bacterial cell death may account for this phenomenon [35]. Meanwhile, previous studies reported that high hydrophobicity and hydrophobic moment values may increase the haemolytic activity [36]. As there was stronger haemolytic activity of OSe than the other analogues, a Pro residue which was restored to form OSf. Interestingly, OSf revealed the broad-spectrum antibacterial property with a dramatic decrease in haemolysis activity. Additionally, the length of OSf (11 amino acids) is much less than that of brevinin-1OS (24 amino acids). Thus, our data suggested that net charge, hydrophobicity and α-helix of peptides are indispensable factors, but a good balance of α-helical propensity and hydrophobicity is necessary. Antimicrobial activity of AMPs could be decreased or even totally lost in different circumstances caused by biological fluid factors like high salts, high temperature, acidic pH and serum [37]. In this study, the antimicrobial effects of brevinin-1OS and designed peptides were reduced to varying degrees under different concentrations of salt concentrations and pH values. The cations (Na^+^ and Mg^2+^) may weaken the binding ability of electrostatic forces between cationic peptides and negatively-charged molecules, such as lipopolysaccharides in the OM and phosphatidylserine in the IM [38,39].

As we knew, cationic peptides may selectively bind to negatively-charged microbial surfaces, including its binding and insertion into the membranes of bacteria cells, bringing about the formation of pores in the membrane, and leakage of cytoplasm, leading to the destruction of the membrane integrity, and ultimately the killing of bacterial cells [40]. Our data revealed that the integrity of both OM and IM were disturbed after treatment with different concentrations of designed peptides. The SEM and TEM results supported that the peptides target on the membrane of bacteria, disrupts the integrity of the membrane, and finally leads to the leakage of cell contents of bacteria. Taken together, we speculated that brevinin-1OS, OSd, OSe and OSf kill *E. coli* and MRSA through the combination of the pore-formation/ion channels (barrel stave model or toroidal model) and the membrane disruption/perturbation modes (carpet-like model) [41].

Likewise, bacteria biofilms are considered to be an enormous threat for the treatment of human infections due to pathogenesis and antibiotic tolerance [42]. As previous reports have shown, compared with planktonic bacteria, sessile bacteria can tolerate much higher doses of antimicrobial agents because of the protection afforded to biofilms [43]. Thus, brevinin-1OS and three peptides (OSd, OSe and OSf), which possessed strong antimicrobial activity, were evaluated for antibiofilm activity. Our results revealed that these peptides also exhibited good antibiofilm activity against select tested microorganisms, however, compared with planktonic cells, these peptides failed to exhibit antibiofilm activity on some selected bacteria. The bacteria in biofilms usually exhibit a different phenotype than planktonic cells and this may explain this phenomenon [44]. Although the mechanism of action of AMPs against biofilms is not clear, one possible explanation is that AMPs have antibiofilm activity by forming pores with the lipid components of the biofilm or passing through the extracellular biofilms or dispersing the biofilms [45,46].

In the pig skin tissue, bacterial morphology, as well as the biofilm, was severely damaged after four days treatment of OSf, suggesting that MRSA biofilms were restrained dramatically. Moreover, the improved survival rate of MRSA-infected larvae in the treatment of OSf was observed in the study. Although the assays in the two models are not able to mimic the real condition in the human body, they could reflect the potential efficacy of the peptides in a more complex environment than the simple in vitro assay. Collectively, the optimised analogue, OSf may indeed be a promising drug candidate for the development of a new therapeutic approach to the treatment of MRSA infections.

## 4. Conclusions

In conclusion, our studies using brevinin-1OS analogues showed that the positive charge in the Ranabox and N-terminus of peptides contributed to their activity. In addition, the balance between positive charge, α-helicity and hydrophobicity, is necessary for maintaining the antimicrobial activity. Among the designed peptides, our results suggested that OSf appears to be a good candidate lead because of its greater antimicrobial activity, good stability and negligible haemolytic activity as well as rapid time-killing against MRSA. The present study also demonstrated that brevinin-1OS, OSd, OSe and OSf exhibited the ability to permeabilise the cell membrane and disrupt membrane integrity, leading to cell death. Furthermore, the skin disinfection test and in vivo evaluation of OSf validated that OSf has a strong ability to inhibit MRSA biofilm infection. Taking all the results together, OSf shows a promising future as a lead compound in developing alternative and effective therapeutic weaponry for medical treatments.

## 5. Materials and Methods

### 5.1. Specimen Preparation and Secretion Harvesting

The collection of the skin secretion from *Odorrana schmackeri* was performed as the previous study [47]. The adults of the specimen were sampled in Fujian Province, China, and washed using deionised water. The frogs were held generally and subjected to a mild electrical stimulation on the dorsal. The skin secretion was washed off and harvested in a clean container, and further snap-frozen in the liquid nitrogen. The procedure of collecting skin secretion was validated by the IACUC of Queen’s University Belfast under the guidelines in the UK Animal (Scientific Procedures) Act 1986 and approved with a project license on 1 March 2011 (code: PPL 2694), which was issued by the Department of Health, Social Services and Public Safety, Northern Ireland.

### 5.2. “Shotgun” Cloning of Biosynthetic Precursor and Identification of the Mature Peptide from the Skin Secretion of Odorrana Schmackeri

The “shotgun” cloning experiment was performed the same as our previous study [47]. Briefly, the mRNA in the skin secretion was extracted using Dynabeads mRNA purification kit. The purified mRNA was subsequently synthesised into 3′-RACE-ready cDNA using the RACE PCR kit. A degenerate sense primer (5′-ATGTTCACCWYRAAGAAATCCMTKYTA-3′; W = A + T, Y = C + T, R=A+G, M = A + C, K= G+T), which was designed to the 5′-UTR or signal peptide region of previously identified skin defensive peptides from close species, was employed to amplify the templates that aimed to obtain the full-length of biosynthetic precursor encoding cDNA. Then, the RACE products were cloned using pGEMT easy vector system and finally sequenced using automatic Sanger DNA sequencer.

In addition, the skin secretion was dissolved and subjected to the reverse phase high-performance liquid chromatography (RP-HPLC) to fractionate the compounds with a gradient elution programme at the flow rate of 1 mL/min. The fraction was collected at 1 min interval. Each fraction was analysed by LCQ fleet ion trap mass spectrometer (Thermo Fisher Scientific, San Jose, CA, USA). The MS/MS spectra were further interpreted through the Sequest algorithm packaged in Protein Discoverer 1.0 software (Thermo Fisher Scientific, San Jose, CA, USA), against the self-defined database that contained the biosynthetic precursor sequences from “shotgun” cloning.

### 5.3. Peptide Design and Synthesis

In this study, the amphipathic α-helical brevinin-1OS was utilized as a template to design peptide analogues. Six peptides were designed, with the purpose of finding short peptides which have high cell selectivity, low haemolysis activity and good stability. OSa, which had an excised Ranabox, was designed first. OSb was further designed by removing the N-terminal hydrophobic amino acid and adding a cationic amino acid (K) at the end of N-terminal of peptides. In order to further increase the activity of peptide, the Gly^4^ was substituted by Lys to form the OSc. OSd was designed by substituting the Pro with Trp firstly, meanwhile, the Ile was replaced by Ala to decrease its hydrophobicity. Primary structures of peptides were analysed using bioinformatics programmes ProtParam (ExPASy Proteomics Server: http://www.expasy.org/tools/protparam.html). The helical wheels of peptides were optimised by using Heliquest software (http://heliquest.ipmc.cnrs.fr). Subsequently, OSe was designed by replacing Val with Trp. Finally, OSf was designed by adding a Pro at position 3 of the N-terminal domain of the peptide. The peptides were amidated to increase their stability. The Fmoc solid phase synthesis method was carried out to produce brevinin-1OS replicates and analogues as previously described [47]. The crude peptides were further purified by RP-HPLC (Amersham Biosciences, Buckinghamshire, UK), coupled with C18 semi-preparative column. The primary structures and purity of peptides in the collected fractions were determined by matrix-assisted laser desorption/ionization time-of-flight mass spectrometer (MALDI-TOF MS, Thermo Fisher Scientific, San Francisco, CA, USA).

### 5.4. Analysis of the Secondary Structure of Synthetic Peptides

Circular dichroism (CD) was employed to determine the secondary structure of the synthetic peptides in the different environment. The peptides were prepared in the aqueous solution, 10 mM NH_4_AC solution (pH 7.4), or the membrane mimicking solution, 50% TFE in 10 mM NH_4_AC, at a concentration of 50 µM. The proportion of helical content of each peptide in the 50% TFE in 10 mM NH_4_AC was calculated using the online server, K2D3 (http://cbdm-01.zdv.uni-mainz.de/~andrade/k2d3/). Physiochemical properties of the helical forming peptides were calculated online by Heliquest (http://heliquest.ipmc.cnrs.fr). Additionally, the secondary structures of peptides were predicted online by I-TASSER Predictions (https://zhanglab.ccmb.med.umich.edu/I-TASSER/).

### 5.5. Antimicrobial Activity Assays

The minimal inhibitory concentrations (MICs) and minimum bactericidal concentrations (MBCs) were evaluated to determine the antimicrobial activity of synthetic peptides. The microorganisms employed in the study are two kinds of Gram-negative bacteria, *Escherichia coli* (NCTC 10418) and *Pseudomonas aeruginosa* (ATCC 27853), three kinds of Gram-positive bacteria, *Staphylococcus aureus* (NCTC 10788), Methicillin-resistant *Staphylococcus aureus* (MRSA, ATCC 12493) and *Enterococcus faecalis* (NCTC 12697) and a yeast, *Candida albicans* (NCPF 1467), as indicated previously [1]. For antimicrobial assays, 5 × 10^5^ CFU/mL of bacterial suspensions were incubated with the concentrations of the peptides (ranging from 1 µM to 512 µM) for 24 h at 37 °C. The sterilised broth medium was used as the positive control and the microorganism cultured in the medium only was treated as the negative control. For the minimum bactericidal concentration (MBC) assay, 20 µL of cell culture suspension was spotted onto a Mueller–Hinton agar (MHA) (Sigma-Aldrich, St. Louis, MO, USA) plate, and incubated at 37 °C for 18 h. The MBC results were defined as the lowest bactericidal concentrations of peptide. The antimicrobial activity of peptides was tested by monitoring the absorbance at 550 nm using an ELISA plate reader (Biolise BioTek EL808, Winooski, VT, USA).

### 5.6. Anti-Biofilm Assays

The anti-biofilm effects of all synthetic peptides were evaluated, which was described in our previous study [48]. The organisms, including *S. aureus* (NCTC 10788), *E. coli* (NCTC 10418), MRSA (ATCC 12493), *E. faecalis* (NCTC 12697) and *P. aeruginosa* (ATCC 27853), were investigated in this study. Gram-positive bacteria were cultured in the TSB broth and Gram-negative bacteria were cultured in the LB broth. The anti-biofilm effects were determined based on the MBIC and MBEC of all the peptides. For the determination of MBIC, 5 × 10^5^ CFU/mL bacterial cultures were incubated with the peptides concentrations (ranging from 1 µM to 512 µM) for 24 h. The sterilised medium and bacterial suspension without any treatment were used as the negative controls and positive controls, respectively. After incubation, wells were rinsed, fixed, stained, washed and dissolved with phosphate buffer saline (PBS), methanol (90%, v/v), crystal violet (0.1%, w/v), sterile deionised water and acetic acid (30%, v/v), respectively. The absorbance of dissolved stain was measured at a 595 nm using a Synergy HT plate reader (Biotech, Minneapolis, MN, USA).

For the MBEC assay, the peptide concentrations were prepared as above. The bacteria were cultured in the 96 well plate for 24–48 h, which allowed the formation of mature biofilm in the plate. Then, the bacterial culture was removed from the plate. The biofilm was further washed to remove the planktonic bacteria using PBS. Then, the fresh broth that contained different peptide concentrations was added in the plate for incubation of 24 h at 37 °C. After incubation, the plate was stained by crystal violet as mentioned above. The definition of MBIC can be given when compared to the negative control group (≥90%, MBIC_90_; ≥50%, MBIC_50_). The definition of the MBEC can be given when compared to the negative control group (≥90%, MBEC_90_; ≥50%, MBEC_50_).

### 5.7. Haemolysis Assays

The haemolytic activity of synthetic peptides was determined using the horse red blood cells that were extracted from the de-fibroblast whole horse blood (TCS Biosciences Ltd. Buckingham, UK). The experiment was performed as described previously [1]. Briefly, the red blood cells were harvested and washed using PBS until all broken red blood cells were removed. Then, a 2% cell suspension was prepared using PBS. The peptide concentrations were set as antimicrobial activity assay and mixed with the red blood cells in the microcentrifuge tubes. After a 2 h-incubation at 37 °C, the tubes were centrifuged and the suspension was transferred into a clear 96 well plate. The absorbance of released haemoglobin in the suspension was detected at 570 nm by a plate reader. Besides, the red blood cells treated with PBS and 1% Triton X-100 were used as the negative and positive controls, respectively. HC_50_ (the concentration of the peptides causing 50% haemolysis of the red blood cells) was calculated by the best-fitted curve. The therapeutic index (TI) was further determined as below:

(1) $$\mathrm{TI}={\mathrm{HC}}_{50}/\sqrt[{n}]{product\;of\;n\;MICs},$$

### 5.8. Measurement of Cytotoxicity

The cell viability of the synthetic peptides was analysed using human dermal microvascular endothelium cells (HMEC). MTT was employed to determine the cell metabolism of the living cells as described previously [1]. The peptide concentrations were applied in 10-fold dilution from 10^−4^ to 10^−9^ M. The cells were treated by the peptides for 24 h at 37 °C. Ten µL of MTT was added in each well of 96 well plate and further incubated for 4 h. The crystalised formazan was dissolved in DMSO and detected in the plate reader at 570 nm.

### 5.9. The Stability Assay of Peptides

The stability of brevinin-1OS, OSd, OSe and OSf were tested as these peptides showed significant antimicrobial activity. The activity of peptides in the different environmental conditions including different concentrations of NaCl (50, 100 and 150 mM) and MgCl_2_ (0.5, 1 and 2 mM), pH (6, 7 and 8), temperature (50 °C, 75 °C and 100 °C) and 10% fetal bovine serum (FBS) solution, was evaluated using the broth microdilution assay as described in the antimicrobial activity assay. For the stability test of peptides in the presence of salt, pH and serum, these conditions were adjusted in the fresh medium that was further used to dilute the bacteria suspension. The peptides were also dissolved in such broth and mixed with the bacteria suspension in the 96 well plate. For the thermal stability test, the peptides were dissolved in the water and incubated at different temperature for 15 min before dosing in the plate. The sterilised broth with corresponding conditions was used as the positive controls, and the bacteria suspension without any treatment was used as the negative controls.

### 5.10. Outer Membrane and Inner Membrane Permeability Assays

The permeabilisation effect of synthetic peptides on the outer membrane (OM) of *E. coli* was determined through the enhanced fluorescence of N-phenyl-1-naphthylamine (NPN) that binds with the compromised OM, as previously described [49]. The concentration of peptides was applied as 1 × MIC, 2 × MIC and 4 × MIC. Positive control and negative control were 10 µg/mL polymyxin B and PBS, respectively.

The permeabilisation effect of synthetic peptides on the inner membrane (IM) of *E. coli* was determined through the detection of the release of β-galactosidase when the cell membrane was damaged [50]. The concentration of peptides was applied as above. The supernatant of bacterial culture was mixed with o-nitrophenyl-β-D-galactopyranosidase (ONPG) in the 96 well plate. The plate was then examined using a microplate reader (Biolise BioTek EL808, Winooski, VT, USA) at excitation and emission wavelengths of 350 nm and 420 nm, respectively. PBS and Triton X-100 were used as a negative control and a positive control, respectively.

### 5.11. The Observation of Bacteria Morphology by Scanning Electron Microscopy (SEM) and Transmission Electron Microscopy (TEM)

The integrity and micromorphology of bacterial cells were assessed by SEM and TEM [40]. Briefly, the log-phase bacterial cells were harvested via centrifugation at 1000× *g* for 10 min. Then, the cells were washed by PBS and resuspended in PBS to an OD_600_ of 0.2. The bacteria suspension was further treated with the peptides at their corresponding MIC for 0.5 h at 37 °C. The control cells were incubated without peptides. After incubation, the bacterial cells were harvested and washed, and subsequently fixed using 2.5% (w/v) glutaraldehyde at 4 °C overnight. Period to the analysis, the fixed cells were dehydrated with ethanol and tert-butanol using a graded series for 15 min. Additionally, the sample was further dehydrated in critical point dryer with liquid CO_2_ and coated with gold–palladium. Hitachi S-4800 scanning electron microscopy (Hitachi S-4800, Hitachi, Tokyo, Japan) was employed to obtain the image of the cell morphology.

Sample preparation for TEM were performed the same way as for SEM. The glutaraldehyde-fixed cells were post-fixed with 1% osmium tetroxide at room temperature for 2 h followed by washing thrice with PBS. The samples were dehydrated similarly and transferred to 1:1 mixtures of absolute acetone and 812 embedding medium for 2–4 h followed by permeation in a mixture (v:v = 2:1) of acetone and 812 embedding medium overnight, and then transferred to absolute 812 embedding medium for 5–8 h. The samples were inserted into an embedding plate followed by incubation at 37 °C overnight. Then, the specimens were incubated in an oven for 48 h at 60 °C. The sectioned samples were stained by uranyl acetate and lead citrate, and subsequently observed by the Hitachi HT7700.

### 5.12. Time-Killing Assays

The time-killing assays were employed to determine the killing kinetics of the peptides on the selected bacteria strains. The experiment was performed as described previously [51]. In brief, the bacteria culture was prepared the same way as the antimicrobial activity assay. A bacteria suspension (OD_600_ = 0.1) was mixed with the peptides at the concentration of their corresponding MICs and 4 × MICs. At different time intervals, 10 µL of aliquots of each sample was transferred into a clean tube and a series of 10-fold dilution was prepared in PBS. A volume of 100 µL of diluted bacteria suspension was spread on an MHA plate, and the number of the bacterial colonies that were formed after an overnight-incubation at 37 °C. The bacteria cultured without any treatment was employed as the negative (growth) control. Three experiments were run in triplicate.

### 5.13. Skin Disinfection Tests

As brevinin-1OSf demonstrated the highest therapeutic index (TI) value among designed short peptides, a skin model was used to determine the anti-infection ability of OSf, according to a previously described technique [52], with some modifications. Specifically, pig skin was sterilized before using. The pig skins were cut into small pieces, and then, a rectangular groove was cut using a skin graft. 100 µL of logarithmic MRSA solution was inoculated on the wound, followed by incubating at 37 °C for 30 min. Subsequently, 10 µL of peptides (1 × MIC) were added on the wound and the pig skins were incubated at 37 °C for 4 days. The pictures of wounds were captured by the digital camera after washing thrice with PBS, fixed with 2.5% (w/v) glutaraldehyde and dehydrated with a graded ethanol series. Finally, the MRSA cells which had attached to the wounded pig skin were visualized by SEM (Hitachi S-4800, Tokyo, Japan).

### 5.14. Evaluation of Anti-MRSA Effects of the Peptides Using the Larvae of Galleria Mellonella

The in vivo study of antimicrobial activity of the peptides was evaluated using the larvae of *Galleria mellonella*, which was described in the previous study with minor modifications [53]. The larvae of *Galleria mellonella* (250 ± 25 mg; Livefood UK Ltd., Rooks Bridge, UK) were selected. The MRSA culture was prepared the same way as the antimicrobial activity assay. Then, MRSA cells were collected and washed, and further diluted to 10^8^ CFU/mL in the saline. Ten µL of MRSA suspension was injected to the larvae and placed in a petri dish. After 2 h, the MRSA infected larvae were administrated with 10 µL of peptides (25 mg/kg, 50 mg/kg) by injection. Saline and vancomycin (20 mg/kg) (Sigma-Aldrich, UK) were used as the negative control and positive control, respectively. Each sample group contained 10 larvae, and the experiment was repeated three times (30 larvae for each group in total). The survival curves were achieved using Prism (Version 6.0; GraphPad Software Inc., San Diego, CA, USA) and the log-rank test was employed to analyze the significance between the different treatments and the controls. * (*p* < 0.05), ** (*p* < 0.01) and *** (*p* < 0.001) represent statistical significance.

## Figures and Tables

**Table 1:** Physico-chemical characteristics of brevinin-1OS and analogues. H represents hydrophobicity; µH represent amphipathicity.

Peptide	Sequence	H	µH	Net Charge	α-Helicity (%)
Brevinin-1OS	FLPGILKVAANVVPGVICAITKKC	0.734	0.386	+3	94.61
OSa	FLPGILKVAANVVPGVI	0.832	0.457	+1	29.6
OSb	FLPGILKVAAK-NH_2_	0.688	0.527	+3	27.5
OSc	FLPKILKVAAK-NH_2_	0.598	0.587	+4	26.9
OSd	FLKWALKVAAK-NH_2_	0.602	0.439	+4	51.66
OSe	FLKWALKWAAK-NH_2_	0.695	0.491	+4	39.09
OSf	FLPKWALKWAAK-NH_2_	0.697	0.370	+4	34.8

**Table 2:** MICs^1^, MBCs^2^, HC_50_^3^ and TI^4^ values of brevinin-1OS and its analogues determined for the six different microorganisms.

Microorganisms		MICs ^1^/MBCs ^2^ (µM)						
		Brevinin-1OS	OSa	OSb	OSc	OSd	OSe	OSf
Gram-positive	*S. aureus*	4/16	>512/>512	256/>512	256/>512	16/32	4/4	8/16
	MRSA	32/32	>512/>512	>512/>512	>512/>512	32/32	8/8	16/64
	*E. faecalis*	8/16	>512/>512	>512/>512	>512/>512	32/64	16/16	32/64
Yeast	*C. albicans*	8/64	>512/>512	512/>512	256/>512	16/32	4/4	8/16
Gam-negative	*E. coli*	128/>512	>512/>512	>512/>512	>512/>512	32/32	16/16	8/16
	*P. aeruginosa*	256/>512	>512/>512	>512/>512	>512/>512	64/64	16/16	64/64
HC_50_^3^		145.8	>512	381.4	>512	>512	64.03	328.3
TI ^4^ (Overall)		5.74	1	0.53	1.59	35.92	7.13	20.52
TI (Gram-positive and yeast)		15.33	1	0.63	2	45.24	9.51	21.61

^1^ MICs is minimal inhibitory concentrations; ^2^ MBCs is minimal bactericidal concentrations; ^3^ HC_50_ is the concentration of the peptides causing 50% haemolysis of the red blood cells. When no 50% haemolysis was observed, at 512 µM, a value of 1024 µM was used to calculate the therapeutic index; ^4^ Therapeutic index (TI) is the ratio of the HC_50_ to the geometric mean of MIC. Larger values indicate greater cell selectivity.

**Table 3:** Antibiofilm activity of peptides against tested bacteria.

Microorganisms	MBIC_50_^1^/MBEC_50_^2^ (µM)				MBIC_90_^3^/MBEC_90_^4^ (µM)			
	Brevinin-1OS	OSd	OSe	OSf	Brevinin-1OS	OSd	OSe	OSf
*S. aureus*	4/32	8/32	4/32	8/32	4/64	32/128	8/32	32/128
MRSA	8/64	32/32	8/32	16/32	32/256	64/256	16/32	64/128
*E. faecalis*	32/256	64/128	64/128	32/128	64/>512	128/256	64/128	64/256
*E. coli*	128/>512	64/>512	16/>512	32/>512	128/>512	128/>512	32/>512	128/>512
*P. aeruginosa*	256/512	128/>512	64/>512	64/>512	256/512	256/>512	128/>512	256/>512

^1^ MBIC_50_ is the minimum biofilm inhibitory concentration required to inhibit the formation of 50% of biofilm; ^2^ MBEC_50_ is the minimum biofilm eradication concentration required to eradicate 50% of the formed biofilm; ^3^ MBIC_90_ is the minimum biofilm inhibitory concentration required to inhibit the formation of 90% of biofilm; ^4^ MBEC_90_ is the minimum biofilm eradication concentration required to eradicate 90% of the formed biofilm.

**Table 4:** MIC values of peptides in the presence of salts (mM), pH, temperature (°C) and serum.

Peptides	Microorganisms	MIC (µM)												
		NaCl (mM)			MgCl_2_ (mM)			pH			Temperature (°C)			Serum (10%)
		50	100	150	0.5	1	2	6	7	8	100	75	50	
Brevinin-1OS	*S. aureus*	8	8	8	4	8	8	8	4	4	4	4	4	4
	MRSA	8	16	32	16	16	16	64	8	4	16	16	16	8
	*E. faecalis*	32	32	64	32	32	32	128	32	16	16	16	16	16
	*C. albicans*	32	32	32	16	16	16	64	16	16	16	16	16	8
	*E. coli*	64	64	64	128	128	128	32	32	128	64	64	64	256
	*P. aeruginosa*	>512	>512	>512	>512	>512	>512	128	256	256	256	256	256	>512
OSd	*S. aureus*	128	256	256	128	128	256	64	32	32	32	32	32	128
	MRSA	512	512	512	512	512	512	256	128	64	128	64	64	128
	*E. faecalis*	512	512	512	512	512	512	256	128	128	256	256	256	>512
	*C. albicans*	128	128	256	64	64	128	64	32	32	128	32	32	64
	*E. coli*	256	256	512	128	256	512	64	64	64	256	64	64	128
	*P. aeruginosa*	256	512	512	256	256	256	128	128	64	128	64	64	256
OSe	*S. aureus*	16	16	16	16	16	16	16	8	4	8	8	8	32
	MRSA	16	16	32	16	16	32	64	16	8	16	16	16	64
	*E. faecalis*	64	64	64	64	64	64	32	64	32	64	64	64	128
	*C. albicans*	16	16	16	8	8	16	16	8	4	8	8	8	16
	*E. coli*	32	32	32	16	64	64	32	16	16	16	16	16	64
	*P. aeruginosa*	64	64	128	64	64	128	32	64	32	32	32	32	128
OSf	*S. aureus*	64	64	64	64	64	64	128	8	16	8	8	8	32
	MRSA	128	256	256	128	128	256	512	16	32	32	16	16	64
	*E. faecalis*	256	256	512	256	256	256	512	32	128	32	32	32	128
	*C. albicans*	64	64	128	32	64	64	128	8	16	16	8	8	32
	*E. coli*	64	128	128	128	128	256	128	8	32	16	8	8	32
	*P. aeruginosa*	128	256	256	128	256	256	128	256	64	64	64	64	64
